# LncRNA PPP1R14B-AS1 Promotes Tumor Cell Proliferation and Migration via the Enhancement of Mitochondrial Respiration

**DOI:** 10.3389/fgene.2020.557614

**Published:** 2020-11-11

**Authors:** Yibin Yang, Yuan Zhang, Lihong Miao, Weijie Liao, Weifang Liao

**Affiliations:** ^1^School of Biology and Pharmaceutical Engineering, Wuhan Polytechnic University, Wuhan, China; ^2^School of Life Sciences, Tsinghua University, Beijing, China

**Keywords:** lncRNA PPP1R14B-AS1, diagnosis, metabolism, proliferation, migration

## Abstract

PPP1R14B-AS1 is an antisense long non-coding RNA with unknown functions. Herein, gene differential analyses were performed using the data of patients with liver cancer and lung adenocarcinoma (LUAD) from The Cancer Genome Atlas database. PPP1R14B-AS1 was found to be upregulated and also overexpressed in 10 other types of cancers. In addition, PPP1R14B-AS1 overexpression was associated with poor overall prognosis in eight cancers. Furthermore, PPPAR14B-AS1 upregulation was positively associated with worsening development of liver and LUAD cancers and related to poor disease-free survival. Gene Ontology and Kyoto Encyclopedia of Genes and Genomes enrichment analyses suggested that PPP1R14B-AS1 strongly participated in regulating cell aerobic respiration processes, such as mitochondrial electron respiration chain and NADH dehydrogenation processes. Cell cytoplasmic and nuclear RNA purification assessment results revealed that PPP1R14B-AS existed in the cell nucleus and cytoplasm. The knockdown of PPP1R14B-AS1 in HepG2 and A549 cells using PPP1R14B-AS1-specific siRNAs decreased mitochondrial respiration as demonstrated by the reduction in basal respiration and ATP production. Moreover, PPP1R14B-AS1 downregulation did not obviously affect cell glycolysis ability. Finally, PPP1R14B-AS1 inhibition inhibited HepG2 and A549 cell migration and proliferation. In summary, our study found for the first time that PPP1R14B-AS1 could be a potential biomarker for cancer diagnosis and that PPP1R14B-AS1 inhibition could be a potentially effective therapy.

## Introduction

Liver cancer (LIHC) and lung cancer rank among the top four most prevalent malignant tumors. They have been emerging as the main causes of human deaths over past decades, thus placing a great burden on society and individuals (Torre et al., 2015; Disease et al., 2017; Bray et al., 2018). Surgery combined with chemoradiotherapy remains the most effective therapy for LIHC and lung cancer. However, the effect of this treatment modality remains limited. One of most important factors for this limitation is the lack of timely diagnosis (Altekruse et al., 2012; Lemjabbar-Alaoui et al., 2015; Zeng et al., 2015). The treatment of LIHC and lung cancer has turned to targeted treatment. The 5-year survival rates of patients have improved but remain inadequate (Hirsch et al., 2017). Therefore, the need for discovery of new diagnostic and prognostic biomarkers for target therapy with increased precision is urgent.

One hundred years ago, Otto Warburg first discovered that under normal oxygen conditions, cancer cells prefer to uptake additional glucose or glutamine for aerobic glycolysis to produce lactate (Warburg, 1956). This phenomenon is called the Warburg effect. Aerobic glycolysis can generate ATP and the necessary substrates for biosynthesis for cell survival. However, it is an uneconomical metabolic process and can only generate two ATP molecules. By contrast, 36 ATP molecules can be synthesized via oxidative phosphorylation (OXPHOS). Glycolytic ATP contribution rates vary from 1.2 to 64% in different tumor cells (Elwood et al., 1963; Kallinowski et al., 1989), suggesting that OXPHOS still plays an important role in tumor cell growth. FDA-approved drugs targeting electron transport chain complexes have shown promising anticancer effects. These drugs include panhematin combined with metformin (complex I), fenofibrate (complex I), and arsenic trioxide (complex IV) (Diepart et al., 2012; Wilk et al., 2015; Lee et al., 2019). However, these drugs remain inadequate, and additional new targets must be discovered.

Long non-coding RNAs (lncRNAs) are RNAs whose transcripts are greater than 200 nucleotides; they lack protein-coding ability or have small peptide-coding potential (Brosnan and Voinnet, 2009; Huang et al., 2017). They mediate many regulatory processes, such as mRNA transcription, mRNA post-transcription, mRNA processing, protein activity, and protein complex organization (Achour and Aguilo, 2018). In recent years, many studies have reported that lncRNAs play roles in the occurrence and development of cancers. For example, lncRNA MAGI2-AS3 functions as a tumor suppressor by recruiting KDM1A and promoting H3K4me2 demethylation of the RACGAP1 promoter in HCC (Pu et al., 2019). TGF-β-activated lncRNA LINC00115 acts as a miRNA sponge to promote GBM tumor growth via the ZNF596/EZH2/STAT3 signaling pathway (Tang et al., 2019). The FOXN3–NEAT1–SIN3A repressor complex promotes EMT and breast cancer cell invasion (Li et al., 2017). Given the important roles of lncRNAs in cancer and the large amount of lncRNAs, finding new lncRNAs associated with tumors is of great value. Thus, this study aimed to find new lncRNAs that participate in cancer development and reveal new insights for cancer drug development.

## Materials and Methods

### Datasets

The RNA-seq datasets of patients with LIHC, lung adenocarcinoma (LUAD), KICH, KIRP, COAD, BLCA, LUSC, PRAD, READ, STAD, THCA, and BRCA were obtained from the TCGA database. The clinical information datasets of patients with LIHC and LUAD were downloaded from the TCGA database.

### Bioinformatics Analyses

Differences in global gene expression levels between the normal tissues and tumor tissues of patients with LIHC/LUAD were analyzed with an R package and *p* < 0.05. The gene copy numbers of the PPP1R14BA-S1 data of 2274 patients with LIHC, 4747 patients with LUAD, 257 patients with bladder cancer, 11244 patients with breast cancer, 2374 patients with prostate gland cancer, 2446 patients with stomach cancer, 3767 patients with colon cancer, 2848 patients with kidney cancer, 422 patients with rectum cancer, and 885 patients with thyroid gland cancer were extracted from Progenetix web resources^1^. Homology among human, rhesus, mouse, dog, elephant, chicken, *Xenopus tropicalis*, zebrafish, and lamprey PPP1R14B-AS1 sequences was analyzed using the UCSC Genome Browser website^2^. Gene expression RNA-seq data for PPP1R14B-AS1 in different human tissues were identified using the GTEx portal website^3^. Survival analysis of patients with LIHC, LUAD, cervical and endocervical cancers (CESC), kidney renal clear cell carcinoma (KIRC), lower grade glioma (LGG), skin cutaneous melanoma (SKCM), and uveal melanoma (UVM) was extracted from GEPIA^4^. Gene coexpression was analyzed with Pearson correlation coefficients using the “cor” function in R. Kyoto Encyclopedia of Genes and Genomes (KEGG) and Gene ontology (GO) enrichment analyses of PPP1R14BA-AS1 and closely related genes were conducted with the R package “clusterProfiler,” and the *p* value cutoff was 0.05.

### Cell Culture and Transfection

HepG2, L02, and A549 cell lines were obtained from the Global Bioresource Center (ATCC) (United States) and grown in Dulbecco’s Modified Eagle Medium (DMEM) (Gibco) with 4.5 g/l D-glucose, 10% fetal bovine serum (FBS) (Gibco), and penicillin/streptomycin (Gibco). Cells were maintained at 37°C in a humidified 5% CO_2_ incubator.

PPP1R14B-AS1-specific siRNAs were designed using siDirect version 2.0 tool^5^ and synthesized by GenePharma Corporation (Shanghai). For PPP1R14B-AS1 knockdown, PPP1R14B-AS1-specific siRNAs and negative siRNA NC were transfected into HepG2 and A549 cells with the transfection reagent Lipofectamine 3000 (Thermo Fisher Scientific) using Opti-MEM (Gibco). The siRNA sequences were as follows: siPPP1R14B-AS1 Target-1 (5′–AGGCTTGAAC AGTCTTCAAAT–3′) and Target-2 (5′–AGGCTGTAACAAAGATTAAAT–3′).

### RNA Extraction and cDNA Synthesis

Cells were harvested, and total RNA was isolated with Trizol (Takara), precipitated with isopropanol, and purified with 75% ethanol. Quality control and concentration check were performed with NanoDrop 2000 (Thermo Fisher Scientific). For RNA reverse-transcription, total RNA was pre-denatured at 65°C for 5 min and then reverse-transcribed with ReverTra Ace qPCR RT Kits (TOYOBO). Reactions were heated to 37°C for 15 min, 98°C for 5 min, and held at 4°C. cDNA was kept at −20°C.

### Fluorescent Quantitative Real-Time PCR

Quantitative real-time PCR (qRT-PCR) was performed with SYBR^®^ Green Real-Time PCR Master Mix (TOYOBO), and ABI 7300 Real-Time PCR system was used for gene expression detection. The reaction steps were as follows: 95°C for 1 min, followed by cycles of 95°C for 15 s and 60°C for 45 s. The results were calculated through the 2^–ΔΔCT^ method, and ACTB expression levels were used as controls. The primer sequences were as follows: PPP1R14B-AS1 forward (5′–TGCTACCAGGCTTGAACAG–3′), reverse (5′–CAGGCACAGAGGAAGACAT–3′); ACTB forward (5′–TGACGTGGACATCCGCAAAG–3′), reverse (5′–CTGGA AGGTGGACAGCGAGG–3′).

### Cell Nuclear and Cytosol RNA Extraction

HepG2, L02, and A549 (200 w) cell nuclear and cytosolic RNA was extracted using a nuclear/cytosol fractionation kit (BioVision, K266-25). Briefly, the cells were lysed with CEBA-A mix buffer and then centrifuged. The supernatant RNA (cytosol RNA) was extracted with Trizol. The pellet was then washed and lysed with Trizol for nuclear RNA extraction.

### Cell Mitochondria Isolation

HepG2 and A549 cell mitochondria were isolated by using Cell Mitochondria Isolation Kit (Beyotime, C3601). In short, 2000 w cells were collected and homogenized. Cell homogenate was centrifuged at 1200 × *g* for 15 min and then supernatant was collected and further centrifuged at 12000 × *g* for 30 min, and the pellet was the mitochondria.

### Western Blot Assay

Protein levels were detected using western blot assay. Cell total protein and mitochondrial protein were harvested with RIPA cell lysis buffer. Antibodies were as follows: ACTB (Proteintech, 60008-1-lg), VDAC1 (Abcam, ab154856), COX IV (CST, 4850S), PARP (CST, 9532S), Cleaved PARP (CST, 5625S), Caspase 3 (CST, 9662S), and Cleaved Caspase 3 (CST, 9664S).

### Seahorse Cell Metabolism Analyses

The cell OXPHOS and cell glycolysis levels of HepG2 and A549 with or without PPP1R14B-AS1 knockdown were measured using a Seahorse XFp analyzer with a Seahorse XFp Cell Mito Stress Test Kit and a Glycolysis Stress Test Kit in accordance with the manufacturers’ instructions.

### Colony Formation Assay

HepG2 and A649 with or without PPP1R14B-AS1 cells were used to perform colony formation assays. In short, 1000 cells were planted in six-well cell culture dishes and incubated with PPP1R14B-AS1-specific siRNAs or negative control siRNA NC for 2 weeks. Then, the cells were fixed and stained with 0.1% crystal violet for 3 min. Cells were calculated using Image J software.

### CCK-8 Assay

Cell proliferation activity was assessed by CCK-8 assay (MCE, K0301) according to the manufacturer’s instructions. Briefly, ∼1000 cells were seeded into 96-well corning cell dished. After they were transfected with PPP1R14B-AS1 specific siRNA for 0, 24, 48, 72, and 96 h, 10 ul CCK-8 solution was added and incubated at 37°C for 1.5 h. The OD values were measured on a microplate reader at 450 nm.

### Cell Migration Analyses

Cell migration was detected via the wound-healing assay. Cells were transfected with PPP1R14B-AS1-specific siRNAs for 24 h and then planted into 24-well cell culture dishes with Ibidi culture inserts (Ibidi, 80209). The inserts were removed when the cells were adherent and then observed under a microscope (0 h). After 24 h, wound-healing degrees were measured with a microscope.

Cell migration assay was also performed through transwell assay. HepG2 and A549 cells (10 w) with or without PPP1R14B-AS1 knockdown were calculated and seeded into the upper chamber of an insert with 100 μl of serum-free DMEM, and 650 μl of DMEM with 20% FBS was added into the lower chamber. After 24 h, the cells on the upper surface of the membrane were gently removed, and the cells on the lower surface were fixed and stained with 0.1% crystal violet. The number of invasive cells was measured and calculated using Image J software.

### Statistical Analyses

The results were shown as median ± interquartile and mean ± standard deviation, and diagrams were plotted with Prism 8.0 and R. Mann–Whitney test, log-rank test, Student’s *t*-test, and one-way ANOVA were performed for statistical comparisons by using SPSS Version 23 and R. Differences were considered significant when *p* < 0.05.

## Results

### PPP1R14B-AS1 Was Abnormally Expressed in LIHC and Lung Adenocarcinoma

**FIGURE 1:**
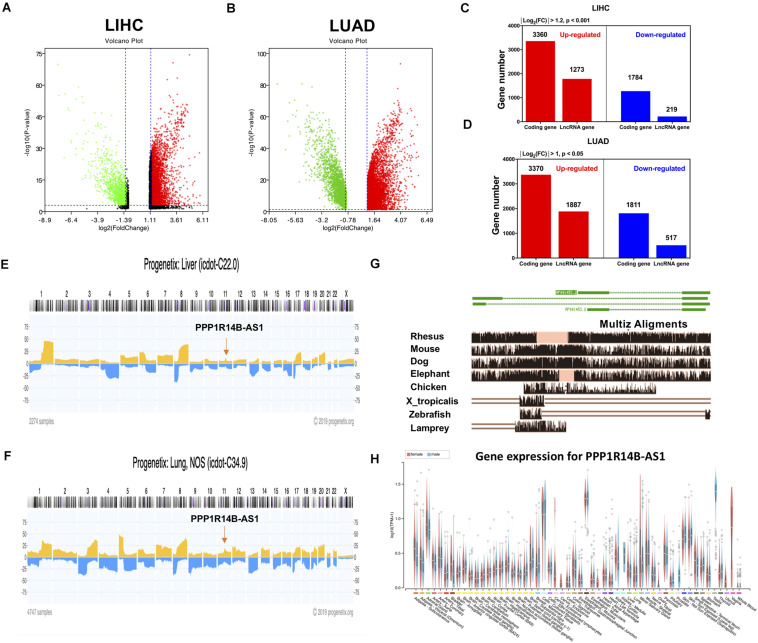
LncRNA PPP1R14B-AS1 was abnormally expressed in LIHC and LUAD. **(A,B)** Volcano plots showing differential gene expression in the cancer tissues of patients with LIHC and LUAD vs. normal tissues, cut-off value of LIHC: absolute Log_2_(fold change) > 1.2 and *p* < 0.001; LUAD: absolute Log2(fold change) > 1 and *p* < 0.05. **(C,D)** Taxonomic statistics of differentially expressed genes in LIHC and LUAD tissues. **(E,F)** Analysis of the copy numbers of PPP1R14B-AS1 across all chromosomes in 2274 LIHC samples and 4747 LUAD samples by Progenetix. **(G)** Eight PPP1R14B-AS1 orthologs from 100 vertebrate species monitored via the Multiz Alignments track in the UCSC Genome Browser. **(H)** PPP1R14B-AS1 expression levels in different normal human tissues provided by GTEx.

RNA-seq data from TCGA were used to conduct gene differential analysis to find new abnormally expressed lncRNAs in LIHC and LUAD. The analysis selectively output 5144 upregulated genes (red, *p* < 0.001) and 1492 downregulated genes (green, *p* < 0.001) in LIHC and 5257 upregulated genes (red, *p* < 0.05) and 2328 downregulated genes (green, *p* < 0.05) in LUAD (Figures 1A,B). Among these differentially expressed genes, 1784 lncRNAs were upregulated in LIHC (Figure 1C) and 1887 lncRNAs were overexpressed in LUAD (Figure 1D).

Herein, we termed an lncRNA that has been named by NCBI database and has never been studied: PPP1R14B-AS. It was located in the 11q13.1 region. By analyzing its position in the genome, we found that it was transcribed from the antisense stand of the PPP1R14B gene. The protein of PPP1R14B was identified to be closed linked to cAMP pathway in Genecard database, which was related to ATP synthesis. Due to the location relationship, we suspect that PPP1R14BA-AS1 might be related to the function of cell energy metabolism. Thus, this lncRNA was selected for further study.

The copy number of genes in this region was upregulated by 10–20% in 2274 patients with LIHC and in 4747 patients with LUAD (Figures 1E,F), which might be associated with PPP1R14B-AS1 overexpression in LIHC and LUAD. According to the UCSC visual database, the homology of PPP1R14B-AS1 decreased from *Rhesus*, mouse, and dog to elephant and showed nearly no homology with 90 other vertebrates (Figure 1G). In contrast to protein-coding genes, lncRNAs are typically tissue-specific. However, PPP1R14B-AS1 was expressed in all of the whole tissues from humans (Figure 1H).

### PPP1R14B-AS1 Was Overexpressed in Many Cancers

**FIGURE 2:**
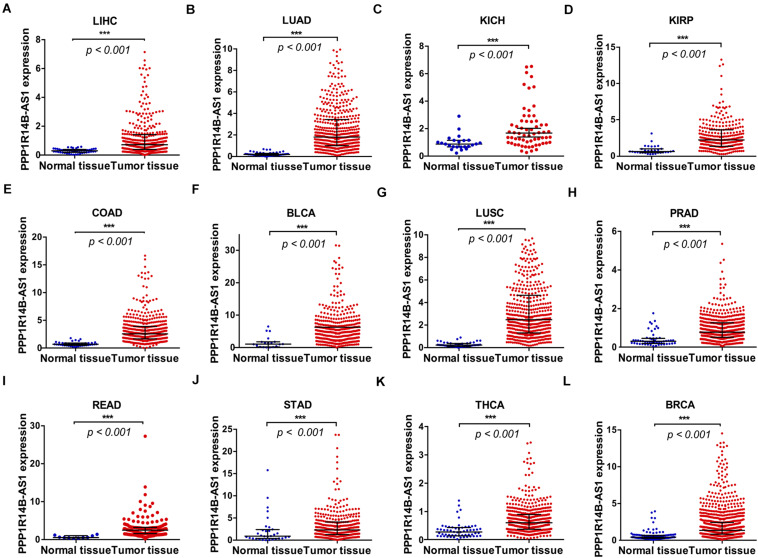
PPP1R14B-AS1 was overexpressed in 12 cancer tissues. PPP1R14B-AS1 RNA-seq FPKM data in the **(A)** normal tissues (*n* = 50) and tumor tissues (*n* = 374) of patients with LIHC. **(B)** Normal tissues (*n* = 59) and tumor tissues (*n* = 533) of patients with LUAD. **(C)** Normal tissues (*n* = 24) and tumor tissues (*n* = 85) of patients with KICH. **(D)** Normal tissues (*n* = 32) and tumor tissues (*n* = 289) of patients with KIRP. **(E)** Normal tissues (*n* = 41) and tumor tissues (*n* = 480) of patients with COAD. **(F)** Normal tissues (*n* = 19) and tumor tissues (*n* = 414) of patients with BLCA. **(G)** Normal tissues (*n* = 49) and tumor tissues (*n* = 502) of patients with LUSC. **(H)** Normal tissues (*n* = 52) and tumor tissues (*n* = 499) of patients with PRAD. **(I)** Normal tissues (*n* = 10) and tumor tissues (*n* = 166) of patients with READ. **(J)** Normal tissues (*n* = 32) and tumor tissues (*n* = 375) of patients with STAD. **(K)** Normal tissues (*n* = 58) and tumor tissues (*n* = 510) of patients with THCA. **(L)** Normal tissues (*n* = 113) and tumor tissues (*n* = 1109) of patients with BRCA. Data were presented as median ± interquartile range and analyzed through Mann–Whitney test, ***p < 0.001.

The PPP1R14B-AS1 RNA-seq FPKM data for 12 different types of cancers from TCGA database were analyzed to assess the expression of PPP1R14B-AS1 in different tumors. Results revealed that PPP1R14BA-S1 was commonly overexpressed in all of the 12 cancer tissues relative to in normal tissues: LIHC tissues (LIHC, *n* = 374, *p* < 0.001, Figure 2A), LUAD tissues (LUAD, *n* = 533, *p* < 0.001, Figure 2B), kidney chromophobe carcinoma tissues (KICH, *n* = 65, *p* < 0.001, Figure 2C), kidney renal papillary cell carcinoma tissues (KIRP, *n* = 289, *p* < 0.001, Figure 2D), colon adenocarcinoma tissues (COAD, *n* = 480, *p* < 0.001, Figure 2E), bladder urothelial carcinoma tissues (BLCA, *n* = 414, *p* < 0.001, Figure 2F), lung squamous cell carcinoma tissues (LUSC, *n* = 502, *p* < 0.001, Figure 2G), prostate adenocarcinoma tissues (PRAD, *n* = 499, *p* < 0.001, Figure 2H), rectum adenocarcinoma tissues (READ, *n* = 166, *p* < 0.001, Figure 2I), stomach adenocarcinoma tissues (STAD, *n* = 375, *p* < 0.001, Figure 2J), thyroid carcinoma tissues (THCA, *n* = 510, *p* < 0.001, Figure 2K), and breast invasive carcinoma tissues (BRCA, *n* = 1109, *p* < 0.001, Figure 2L). In addition, we found that the copy number of genes in 11q13.1 region was upregulated by 10–25% in 257 patients with BLCA, 11244 patients with BRCA, 2374 patients with PRAD, 2446 patients with STAD, and 3767 patients with COAD (Supplementary Figures S1A–E). However, no obvious gene copy number gain was found in kidney cancer patients, rectum cancer patients, or thyroid gland cancer patients (Supplementary Figures S1F–H). These data suggest that PPP1R14B-AS1 upregulation might also be associated with the increase in gene copy number in BLCA, BRCA, PRAD, STAD, and COAD patients. In short, these results demonstrated that PPP1R14B-AS1 upregulation could be a potential biomarker for the diagnosis of these cancers.

### PPP1R14B-AS1 Overexpression Was Associated With Poor Overall Survival

**FIGURE 3:**
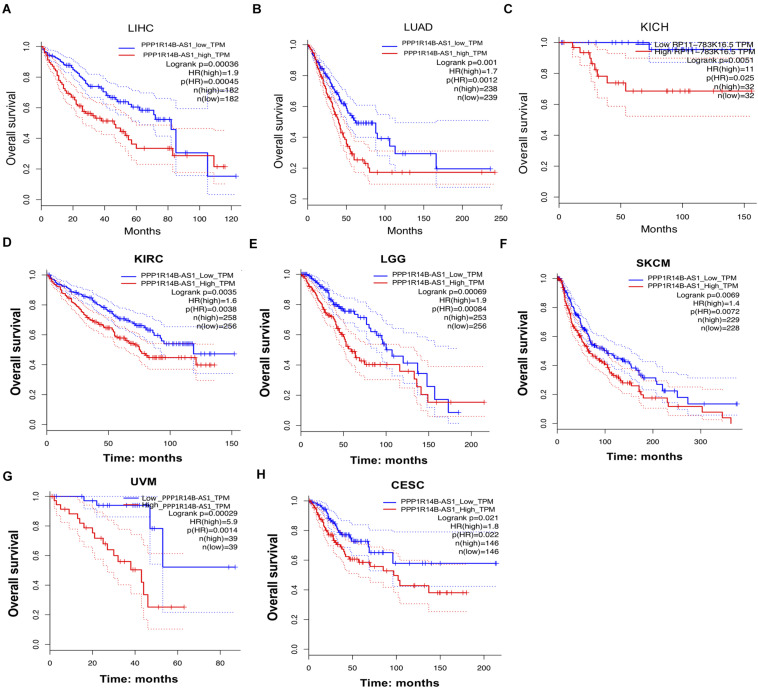
PPP1R14B-AS1 upregulation was related to poor survival in several cancers. **(A)** Patients with LIHC were divide into two groups in accordance with the median of the expression value of PPP1R14B-AS1 (PPP1R14B-AS1_low and PPP1R14B-AS1_high), and then Kaplan–Meier survival curves were constructed to analyze the overall survival (OS) of the two groups by GEPIA gene analysis tool (http://gepia.cancer-pku.cn/detail.php?gene=&clicktag=survival). Hazards Ratio (HR) was calculated based on Cox PH Model. **(B–H)** Patients with LUAD, KICH, KIRC, LGG, SKCM, UVM, or CESC were divided into the PPP1R14B-AS1_low and PPP1R14BA-AS1_high groups according to the median value of PPP1R14B-AS1 expression, respectively, and then Kaplan–Meier survival curves were constructed to analyze the OS of the two groups by using the GEPIA gene analysis tool. Data were analyzed through the log-rank test.

The clinical information of the patients was used for analysis to evaluate the relationship between PPP1R14B-AS1 expression and overall survival (OS). First, the patients were stratified into two groups in accordance with the median values of PPP1R14B-AS1 RNA expression (PPP1R14B-AS1_high and PPP1R14B-AS1_low). Second, survival curves were plotted and determined through Kaplan–Meier analysis by GEPIA gene analysis tool. The LIHC patients with high PPP1R14B-AS1 expression showed poor OS (*n* = 364, HR [high] = 1.9, *p*[HR] = 0.00045, log rank *p* = 0.00036, Figure 3A). LUAD patients with high PPP1R14B-AS1 expression also showed poor OS (*n* = 477, HR [high] = 1.7, *p*[HR] = 0.0012, log rank *p* = 0.001, Figure 3B). Moreover, KICH patients with high PPP1R14B-AS1 expression exhibited poor OS (*n* = 64, HR [high] = 11, *p*[HR] = 0.025, log rank *p* = 0.0051, Figure 3C). However, nine other types of cancer patients with upregulated PPP1R14B-AS1 expression showed no obvious difference in OS (Supplementary Figures S2A–I). We also analyzed other types of cancer patients without PPP1R14B-AS1 upregulated (vs. the normal) to study whether PPP1R14B-AS1 could affect the prognosis. Patients with KIRC, brain in LGG, SKCM, and UVM or CESC and high PPP1R14B-AS1 expression had poor OS (Figures 3D–H). These results demonstrated that activated PPP1R14B-AS1 expression was associated with poor survival in these eight types of cancer.

### PPP1R14B-AS1 Functioned in LIHC and LUAD Progression

**FIGURE 4:**
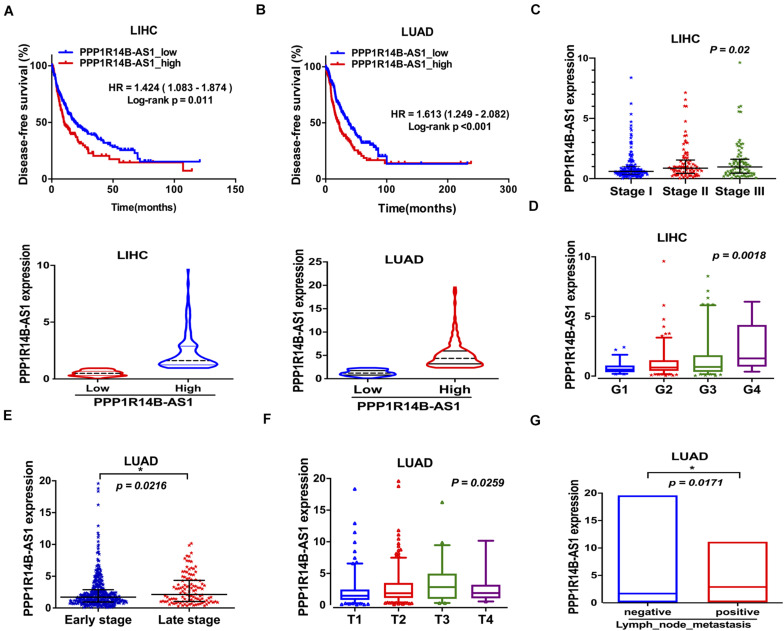
PPP1R14B-AS1 participated in LIHC and LUAD progression. **(A,B)** Patients with LIHC or LUAD were divided into two groups in accordance with PPP1R14B-AS1 RNA-seq FPKM values, and Kaplan–Meier survival analyses were conducted to analyze disease-free survival. HR values were calculated with the R package “survival,” and data were analyzed through log-rank test. **(C)** PPP1R14B-AS1 expression in patients with Stage I (*n* = 171), Stage II (*n* = 86), and Stage III (*n* = 85) LIHC, one-way ANOVA. **(D)** PPP1R14B-AS1 expression in patients with tumor grade 1 (*n* = 55), grade 2 (*n* = 177), grade 3 (*n* = 122), and grade 4 (*n* = 12) LIHC, one-way ANOVA. **(E)** PPP1R14B-AS1 expression in patients with early stage (Stage I + Stage II, *n* = 395) and late-stage (Stage III + Stage IV, *n* = 110) LUAD, Mann–Whitney test. **(F)** PPP1R14B-AS1 expression in primary tumors with different sizes and extents in patients with LUAD (T1, *n* = 168; T2, *n* = 276; T3, *n* = 47; T4, *n* = 19), one-way ANOVA. **(G)** PPP1R14B-AS1 expression in patients with negative (*n* = 330) and positive (*n* = 171) lymph node metastasis, Mann–Whitney test. *p < 0.05.

We analyzed whether PPP1R14B-AS1 participates in LIHC and LUAD progression. After their first surgical treatment, patients with LIHC and high PPP1R14B-AS1 expression showed poor disease-free survival (DFS) (*n* = 353, HR = 1.424, 95%CI: 1.083–1.874, *p* = 0.011, Figure 4A), and patients with LUAD and PPP1R14B-AS1 overexpression also showed poor DFS (*n* = 476, HR = 1.613, 95% CI: 1.249–2.082, *p* < 0.001, Figure 4B). During disease progression, PPP1R14B-AS1 expression changed, exhibiting an upward trend from Stage I to Stage III in LIHC (*p* = 0.0168, Figure 4C). PPP1R14B-AS1 expression in the late stage of the disease (Stage III + Stage IV) was also upregulated compared with that in the early stage of the disease (Stage I + Stage II) (*p* = 0.0216, Figure 4D). Moreover, PPP1R14B-AS1 expression level was highly inversely related to the grade of tumor cell differentiation in LIHC, suggesting that PPP1R14B-AS1 might be one of the factors that aggravated the degree of malignancy (Figure 4E). In addition, PPP1R14B-AS1 expression varied from the primary tumor range and showed a positive relation (Figure 4F). PPP1R14B-AS1 overexpression was also associated with lymph node metastasis in LUAD (Figure 4G). These data indicated that PPP1R14B-AS1 played multiple roles in LIHC and LUAD progression.

### PPP1R14B-AS1 and Its Co-expressed Genes Participated in Mitochondrial Function and Tumor Metastasis Regulation

**FIGURE 5:**
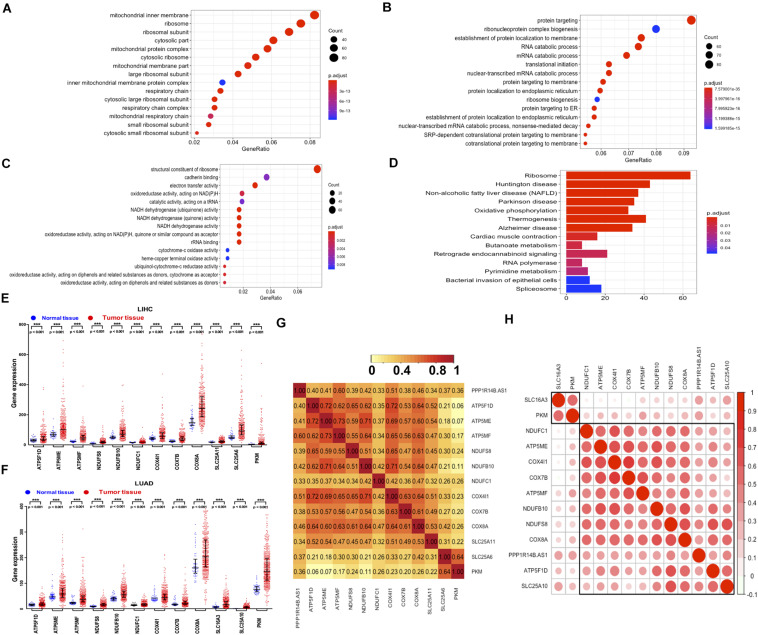
PPP1R14B-AS1 and its correlated genes participated in regulating mitochondrial function and tumor metastasis. **(A–C)** GO enrichment analyses of PPP1R14B-AS1 and its co-expressed genes in LIHC, cut-off values as absolute Pearson correlation coefficients > 0.3 and *p* < 0.05. **(D)** KEGG enrichment analyses of PPP1R14B-AS1 and its co-expressed genes in LIHC, cut-off values as absolute Pearson correlation coefficients > 0.3 and *p* < 0.05. **(E,F)** Expression of typical genes related to PPP1R14B-AS1 in patients with LIHC or LUAD, Mann–Whitney test. ***p < 0.001. **(G,H)** Correlation heatmaps of typical genes related to OXPHOS and associated with PPP1R14B-AS1 in LIHC and LUAD.

PPP1R14B-AS1 and its co-expressed genes were analyzed to reveal the potential biological function of PPP1R14B-AS1. Correlated genes were identified using Pearson correlation analysis by using the RNA-seq FPKM data of LIHC and the *cor* function in R enrichment. The selected cut-off values were set as absolute Pearson correlation coefficient > 0.3 and *p* < 0.05. Gene ontology (GO) and KEGG enrichment analyses were performed by using the filtered genes. GO analysis revealed that cellular components associated with the mitochondrial membrane, mitochondrial protein complex, and respiratory chain were highly enriched (Figure 5A). In addition, the high enrichment shown by the biological processes of RNA catabolism and translational initiation suggested that PPP1R14B-AS1 was involved in gene transcription and post-transcriptional regulation processes (Figure 5B). Moreover, the GO terms in molecular function that were greatly enriched were cadherin binding, electron transfer activity, and NADH dehydrogenase activity (Figure 5C). KEGG enrichment further confirmed the potential function of PPP1R14B-AS1 in OXPHOS pathway regulation (Figure 5D). Furthermore, the representative expressed genes related to ATP synthesis and NADH oxidation in LIHC tissues were all overexpressed relative to those in normal tissues (Figure 5E). These genes were also proved to be upregulated in LUAD tumor tissues (Figure 5F). Meanwhile, all of these genes were positively correlated with PPP1R14B-AS1 in LIHC (Figure 5G) and LUAD (Figure 5H). These data indicated that PPP1R14B-AS1 was closely linked to mitochondrial function and tumor metastasis.

### PPP1R14B-AS1 Regulated Mitochondrial Respiration

**FIGURE 6:**
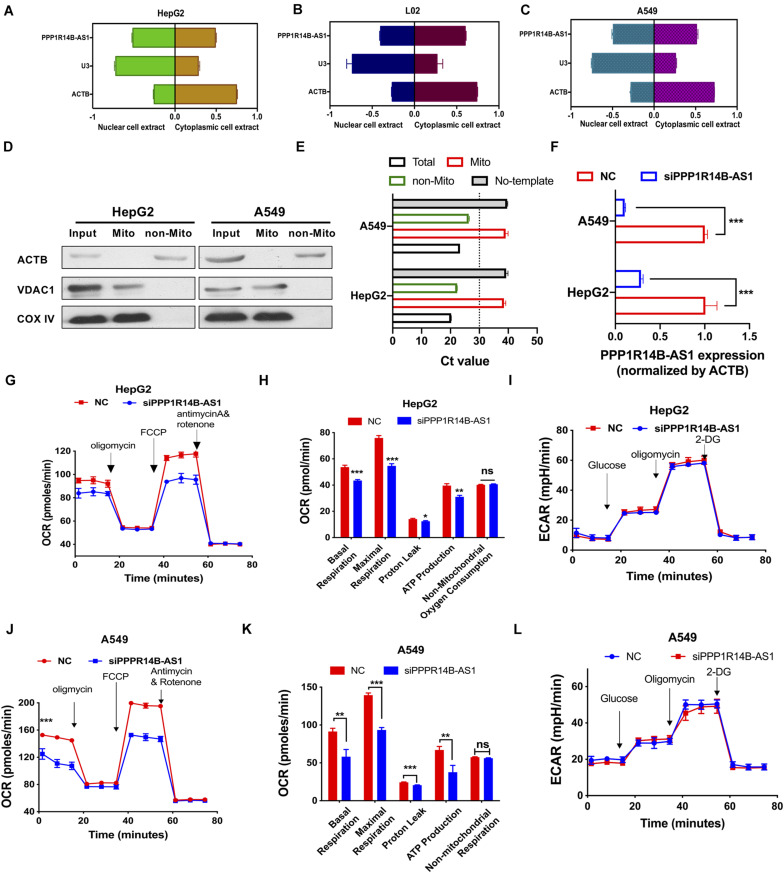
PPP1R14B-AS1 regulated mitochondrial OXPHOS. **(A–C)** qRT-PCR detection of PPP1R14B-AS1 distributions in HepG2, L02, and A549 cells; U3 was used as a nuclear control, whereas ACTB was used as a cytosolic control. **(D)** Mitochondria of HepG2 and A549 cells was isolated, and then western blot assay was carried out, ACTB as non-mitochondrial control while VDAC1 and COXIV as mitochondrial control. **(E)** qRT-PCR assay was performed to detect the expression of PPP1R14BA-AS1 in the mitochondrial components of HepG2 and A549 cells. **(F)** PPP1R14B-AS1 knockdown efficiency in HepG2 and A549 cells, Student’s *t*-test. **(G,H)** Seahorse XFp Cell Mito Stress Test assays were performed on HepG2 cells with or without PPP1R14B-AS1 knockdown, and cell basal respiration, maximal respiration, proton leak, ATP production, and non-mitochondrial oxygen consumption were calculated and analyzed through Student’s *t*-test. **(I)** Seahorse XFp Cell Glycolysis Stress Test assays were conducted using HepG2 cells with or without PPP1R14B-AS1 knockdown. **(J,K)** Seahorse XFp Cell Mito Stress Test assays were performed on A549 cells with or without PPP1R14B-AS1 knockdown, and cell basal respiration, maximal respiration, proton leak, ATP production, and non-mitochondrial oxygen consumption were calculated and analyzed by Student’s *t*-test. **(L)** Seahorse XFp Cell Glycolysis Stress Test assays detected ECAR in A549 cells with or without PPP1R14B-AS1 knockdown. *p < 0.05, **p < 0.01, ***p < 0.001.

On the basis of the clues provided by the above analyses, we performed *in vitro* experiments to verify the biological functions of PPP1R14B-AS1. Cell cytoplasm and nuclear RNA purification experiments showed that PPP1R14B-AS1 was distributed in the cell nuclei and cytoplasm of the human LIHC cell line HepG2 (ACTB was used as the cytoplasmic control, and U3 was used as the nuclear control) (Figure 6A). Similar results for the human normal liver cell line L02 (Figure 6B) and the human LUAD cell line A549 (Figure 6C) were obtained. These results further supported the GO analyses results and were consistent with previous reports showing that lncRNAs regulate gene transcription in the cell nucleus and post-transcriptional processes, such as translation and protein modification, in the cytoplasm. We also detected whether PPP1R14BA-AS1 located on the mitochondrial components. The result revealed that it wasn’t in HepG2 or A549 cells (Figures 6D,E). Further, PPP1R14B-AS1 specific siRNA was designed and transfected into HepG2 and A549. The qRT-PCR results showed great knockdown efficiency (Figure 6F). The Seahorse XFp Cell Mito Stress Test assay elucidated that oxygen consumption rate (OCR) was reduced in HepG2 cells with PPP1R14B-AS1 knockdown. Furthermore, when PPP1R14B-AS1 was silenced, maximal respiration, proton leak, and ATP production all decreased, but non-mitochondrial oxygen consumption did not change (Figures 6G,H). However, the Seahorse XFp Glycolysis Stress Test revealed that PPP1R14B-AS1 did not play any role in glycolysis (Figure 6I). In addition, PPP1R14B-AS1 knockdown decreased OCR and ATP production in A549 cells (Figures 6J,K). Likewise, PPP1R14B-AS1 downregulation had no effect on glycolysis in A549 cells (Figure 6L). These data indicated that PPP1R14B-AS1 enhanced mitochondrial respiration and did not change glycolysis levels in HepG2 and A549 cells.

### PPP1R14B-AS1 Knockdown Inhibited Tumor Cell Proliferation and Migration

**FIGURE 7:**
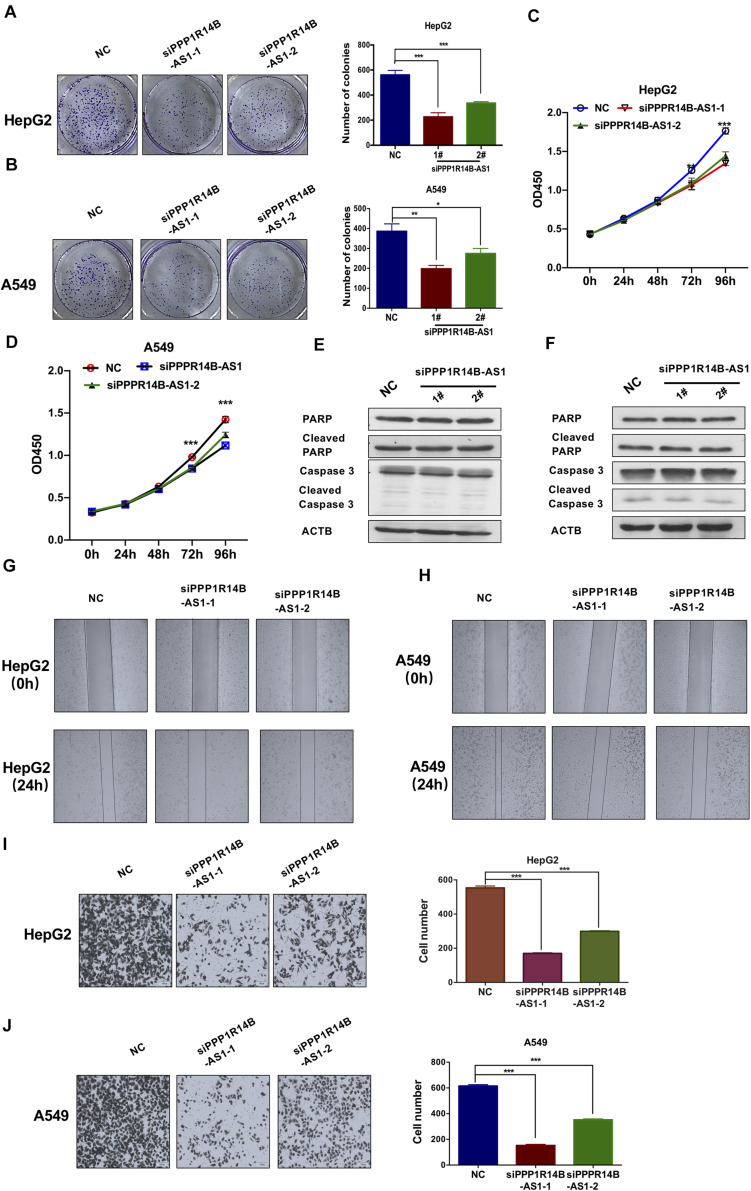
PPP1R14B-AS1 knockdown inhibited tumor cell proliferation and migration. **(A)** Colony formation assay of control HepG2 cells (NC) or PPP1R14B-AS1 knockdown cells (siPPP1R14B-AS1-2 and siPPP1R14B-AS1-2). Cell colonies were counted using Image J software, Student’s *t*-test. **(B)** Colony formation assay was conducted using A549 cells with or without PPP1R14B-AS1 knockdown, and results were analyzed by counting the cell colonies using Image J software, Student’s *t*-test. **(C,D)** CCK-8 assays were preformed to assess the proliferation activity of HepG2 and A549 cells with or without PPP1R14B-AS1 knockdown. **(E,F)** HepG2 and A549 cells with or without PPP1R14B-AS1 knockdown for 72 h, and then cells were lysed and key proteins (Caspase 3 and PARP) related to apoptosis were detected by western blot. **(G,H)** Wound-healing assays showed that PPP1R14B-AS1 knockdown inhibited HepG2 and A549 cell migration. **(I,J)** Transwell assays revealed that PPP1R14B-AS1 inhibition restrained HepG2 and A549 cell invasion. Cell numbers were analyzed using Image J software, Student’s *t*-test. *p < 0.05, **p < 0.01, ***p < 0.001.

Tumor cell growth inhibition and migration inhibition are the two most effective ways to treat tumors. We conducted colony formation assays to investigate the effect of PPP1R14B-AS1 on cell proliferation. The assays revealed that the loss of function of PPP1R14B-AS1 inhibited HepG2 and A549 cell growth (Figures 7A,B). CCK-8 assays were also performed to assess the proliferation activity of HepG2 and A549 cells with or without PPP1R14B-AS1 knockdown. The results showed that the proliferation activity of both HepG2 and A549 cells were significantly reduced after PPP1R14B-AS1 knockdown for 72 h (Figures 7C,D). To further investigate whether PPP1R14B-AS1 affected HepG2 and A549 cells apoptosis, we detected key proteins (Caspase 3 and PARP) levels related to apoptosis after PPP1R14BA-AS1 knockdown for 72 h in HepG2 and A549 cells. Western blot assays revealed that PPP1R14B-AS1 inhibition didn’t change the apoptosis levels (Figures 7E,F).

Considering the clues offered by GO enrichment analysis, we implemented wound-healing assays to study if PPP1R14B-AS1 plays a role in HepG2 and A549 cell migration. The assays showed that HepG2 and A549 cell migration was markedly inhibited when PPP1R14B-AS1 was knocked down (Figures 7G,H). Moreover, transwell assays revealed that PPP1R14B-AS1 inhibition could markedly restrain HepG2 and A549 cell invasion (Figures 7I,J). These results indicated that the loss of function of PPP1R14B-AS1 remarkably repressed HepG2 and A549 cell migration. Taken together, these data demonstrated that PPP1R14B-AS1 inhibition inhibited HepG2 and A549 cell proliferation and migration.

## Discussion

Cancer is now becoming the second most lethal disease worldwide (Collaborators, 2018). LIHC and LUAD are two common fatal cancers with the top five highest morbidity and mortality rates (Bray et al., 2018). LncRNAs are of great value for cancer diagnosis and treatment (Gupta et al., 2010; Keniry et al., 2012; Wang et al., 2017; Chen et al., 2018; Xu et al., 2019). In this study, the results of gene differential analysis performed using the RNA-seq data of LIHC and LUAD obtained from TCGA highlighted lncRNA PPP1R14B-AS1, which had never been reported before. PPP1R14B-AS1 was identified to be overexpressed in 12 cancers, including LIHC and LUAD, indicating that PPP1R14B-AS1 could be a good diagnostic biomarker. Data from the Progenetix database revealed that PPP1R14B-AS1 was located in a region where the gene copy number was upregulated by 10–15% in LIHC and LUAD. The localization of PPP1R14B-AS1 in this region might be one of the reasons that PPP1R14B-AS1 expression was upregulated in LIHC and LUAD. Moreover, PPP1R14B-AS1 upregulation was associated with poor OS in eight cancers, including LIHC and LUAD.

We subjected clinical information to specific analyses to investigate the roles of PPP1R14B-AS1 in LIHC and LUAD progression. We found that high PPP1R14B-AS1 expression was related to the poor DFS of patients with LIHC and LUAD. This relationship suggested that PPP1R14B-AS1 could be a potential target for the development of antitumor drugs for LIHC and LUAD. In addition, high PPP1R14B-AS1 expression was accompanied by disease progression from the early stage to the late stage. Moreover, high PPP1R14B-AS1 expression was associated with the high tumor malignancy degree of LIHC. We also found that PPP1R14B-AS1 in patients with LUAD and lymph node metastasis was overexpressed relative to that in patients with LUAD and without lymph node metastasis. For the first time, these findings provided information showing that PPP1R14B-AS1 was upregulated in LIHC and LUAD progression.

Glycolysis had become a superstar process in cancer research since the Warburg effect was first proposed. Warburg theory states that the irreversible damage of mitochondrial function in tumor cells is the main reason for the selection of glycolysis (Warburg, 1956). However, this view is challenged by many studies that demonstrated through observations that the enzymes for the tricarboxylic acid cycle function normally in many tumor cells and mitochondrial metabolism plays an important role in tumorigenesis (Wenner et al., 1952; Weinberg et al., 2010). Herein, using data from TCGA, the genes that were co-expressed with PPP1R14B-AS1, were selected and used to conduct GO and KEGG enrichment analyses. These analyses showed that these genes were highly related to mitochondrial membrane component, mitochondrial respiratory complex, OXPHOS, and NADH dehydrogenase activity. In addition, we found that many genes associated with ATP synthesis, such as ATP5F1D, ATP5ME, and ATP5MF, and genes associated with NADH oxidation, such as NDUFS8, NDUFB10, and NDUFC1, were overexpressed in LIHC and LUAD tumor tissues. Moreover, these genes were positively related to PPP1R14B-AS1. When PPP1R14B-AS1 was silenced using specific targeted siRNA, HepG2 and A549 cells exhibited reduced mitochondrial respiration as demonstrated by their decreased OCR and ATP production but unchanged non-mitochondrial oxygen consumption and glycolysis.

The most common way to treat tumors is to inhibit tumor cell growth and metastasis. Herein, we found that PPP1R14B-AS1 inhibition could markedly decrease HepG2 and A549 cell proliferation as demonstrated by CCK8 and colony formation assays. PPP1R14B-AS1 knockdown didn’t change the apoptosis of HepG2 and A549 cells. Moreover, transwell and wound-healing assays proved that PPP1R14B-AS1 knockdown could inhibit HepG2 and A549 cell migration. The mechanism of this effect might be OXPHOS repression after PPP1R14B-AS1 downregulation. Our study is the first to highlight PPP1R14B-AS1, a new lncRNA that functions as an oncogene. The inhibition of this lncRNA can repress growth and migration by reducing mitochondrial respiration. Our study provides new insights for the development of therapeutic drugs for LIHC and LUAD in the future.

## Data Availability Statement

The RNA-seq datasets of patients with LIHC, LUAD, KICH, KIRP, COAD, BLCA, LUSC, PRAD, READ, STAD, THCA, and BRCA were obtained from the TCGA database. The clinical information datasets of patients with LIHC, LUAD, and PAAD were downloaded from the TCGA database.

## Author Contributions

WfL and WjL conceived and designed the experiments. YbY, YZ, WjL, and WfL performed the experiments. WjL, WfL, and LhM analyzed the data. WfL, WjL, and YbY contributed to the materials/reagents/analysis tools. WfL wrote the manuscript. All authors contributed to the article and approved the submitted version.

## Conflict of Interest

The authors declare that the research was conducted in the absence of any commercial or financial relationships that could be construed as a potential conflict of interest.

## Footnotes

1 http://www.progenetix.org

2 http://www.genome.ucsc.edu

3 https://www.gtexportal.org

4 http://gepia.cancer-pku.cn

5 http://sidirect2.rnai.jp

## Supplementary Material

Supplementary Figure 1 The copy numbers of 11q13.1 across all chromosomes in different cancer samples. **(A–E)** Analysis of the copy numbers of PPP1R14B-AS1 across all chromosomes in 257 patients with BLCA, 11244 patients with BRCA, 2374 patients with PRAD, 2446 patients with STAD, and 3767 patients with COAD by Progenetix. **(F–H)** Analysis of the copy numbers of PPP1R14B-AS1 across all chromosomes in Kidney cancer patients, Rectum cancer patients and Thyroid gland cancer patients by Progenetix.

Supplementary Figure 2 PPP1R14B-AS1 upregulation showed no correlation with overall survival (OS) in several cancers. **(A–I)** Patients with KIRP **(A)**, COAD **(B)**, BLCA **(C)**, LUSC **(D)**, PRAD **(E)**, READ **(F)**, STAD **(G)**, THCA **(H)**, and BRCA **(I)** were divided into two groups in accordance with the median of the expression value of PPP1R14B-AS1 (PPP1R14B-AS1_low and PPP1R14B-AS1_high) respectively, and then Kaplan–Meier survival curves were constructed to analyze the OS of the two groups by GEPIA gene analysis tool (http://gepia.cancer-pku.cn/detail.php?gene=&clicktag=survival). Hazards Ratio (HR) was calculated based on Cox PH Model.

## References

[B1] Achour C. Aguilo F. (2018). Long non-coding RNA and Polycomb: an intricate partnership in cancer biology. *Front. Biosci.* 23:2106–2132. 10.2741/4693 29772549

[B2] Altekruse S. F. McGlynn K. A. Dickie L. A. Kleiner D. E. (2012). Hepatocellular carcinoma confirmation, treatment, and survival in surveillance, epidemiology, and end results registries, 1992-2008. *Hepatology* 55 476–482. 10.1002/hep.24710 21953588 PMC3868012

[B3] Bray F. Ferlay J. Soerjomataram I. Siegel R. L. Torre L. A. Jemal A. (2018). Global cancer statistics 2018: GLOBOCAN estimates of incidence and mortality worldwide for 36 cancers in 185 countries. *CA Cancer J. Clin.* 68 394–424. 10.3322/caac.21492 30207593

[B4] Brosnan C. A. Voinnet O. (2009). The long and the short of noncoding RNAs. *Curr. Opin. Cell Biol.* 21 416–425. 19447594 10.1016/j.ceb.2009.04.001

[B5] Chen J. F. Wu P. Xia R. Yang J. Huo X. Y. Gu D. Y. (2018). STAT3-induced lncRNA HAGLROS overexpression contributes to the malignant progression of gastric cancer cells via mTOR signal-mediated inhibition of autophagy. *Mol. Cancer* 17:6. 10.1186/s12943-017-0756-y PMC5767073 29329543

[B6] Collaborators G. S. (2018). Measuring progress from 1990 to 2017 and projecting attainment to 2030 of the health-related Sustainable Development Goals for 195 countries and territories: a systematic analysis for the Global Burden of Disease Study 2017. *Lancet* 392 2091–2138. 30496107 10.1016/S0140-6736(18)32281-5 PMC6227911

[B7] Diepart C. Karroum O. Magat J. Feron O. Verrax J. Calderon P. B. (2012). Arsenic trioxide treatment decreases the oxygen consumption rate of tumor cells and radiosensitizes solid tumors. *Cancer Res.* 72 482–490. 10.1158/0008-5472.can-11-1755 22139377

[B8] Disease G. B. D. Injury I. Prevalence C. (2017). Global, regional, and national incidence, prevalence, and years lived with disability for 328 diseases and injuries for 195 countries, 1990-2016: a systematic analysis for the Global Burden of Disease Study 2016. *Lancet* 390 1211–1259. 28919117 10.1016/S0140-6736(17)32154-2 PMC5605509

[B9] Elwood J. C. Lin Y. C. Cristofalo V. J. Weinhouse S. Morris H. P. (1963). Glucose Utilization in Homogenates of the Morris Hepatoma 5123 and Related Tumors. *Cancer Res.* 23 906–913. 14079156

[B10] Gupta R. A. Shah N. Wang K. C. Kim J. Horlings H. M. Wong D. J. (2010). Long non-coding RNA HOTAIR reprograms chromatin state to promote cancer metastasis. *Nature* 464 1071–1076. 10.1038/nature08975 20393566 PMC3049919

[B11] Hirsch F. R. Scagliotti G. V. Mulshine J. L. Kwon R. Curran W. J. Wu Y. L. (2017). Lung cancer: current therapies and new targeted treatments. *Lancet* 389 299–311. 10.1016/s0140-6736(16)30958-8 27574741

[B12] Huang J. Z. Chen M. Gao X. C. Zhu S. Huang H. Hu M. (2017). A Peptide Encoded by a Putative lncRNA HOXB-AS3 Suppresses Colon Cancer Growth. *Mol. Cell* 68 171–184. 10.1016/j.molcel.2017.09.015 28985503

[B13] Kallinowski F. Schlenger K. H. Runkel S. Kloes M. Stohrer M. Okunieff P. (1989). Blood flow, metabolism, cellular microenvironment, and growth rate of human tumor xenografts. *Cancer Res.* 49 3759–3764. 2736517

[B14] Keniry A. Oxley D. Monnier P. Kyba M. Dandolo L. Smits G. (2012). The H19 lincRNA is a developmental reservoir of miR-675 that suppresses growth and Igf1r. *Nat. Cell Biol.* 14 659–665. 10.1038/ncb2521 22684254 PMC3389517

[B15] Lee J. Yesilkanal A. E. Wynne J. P. Frankenberger C. Liu J. Yan J. (2019). Effective breast cancer combination therapy targeting BACH1 and mitochondrial metabolism. *Nature* 568 254–258. 10.1038/s41586-019-1005-x 30842661 PMC6698916

[B16] Lemjabbar-Alaoui H. Hassan O. U. Yang Y. W. Buchanan P. (2015). Lung cancer: Biology and treatment options. *Biochim. Biophys. Acta* 1856 189–210. 26297204 10.1016/j.bbcan.2015.08.002 PMC4663145

[B17] Li W. Zhang Z. Liu X. Cheng X. Zhang Y. Han X. (2017). The FOXN3-NEAT1-SIN3A repressor complex promotes progression of hormonally responsive breast cancer. *J. Clin. Invest.* 127 3421–3440. 10.1172/jci94233 28805661 PMC5669564

[B18] Pu J. Wang J. Wei H. Lu T. Wu X. Wu Y. (2019). lncRNA MAGI2-AS3 Prevents the Development of HCC via Recruiting KDM1A and Promoting H3K4me2 Demethylation of the RACGAP1 Promoter. *Mol. Ther. Nucleic Acids* 18 351–362. 10.1016/j.omtn.2019.08.020 31629962 PMC6807294

[B19] Tang J. Yu B. Li Y. Zhang W. Alvarez A. A. Hu B. (2019). TGF-beta-activated lncRNA LINC00115 is a critical regulator of glioma stem-like cell tumorigenicity. *EMBO Rep.* 20:e48170. 10.15252/embr.201948170 PMC6893290 31599491

[B20] Torre L. A. Bray F. Siegel R. L. Ferlay J. Lortet-Tieulent J. Jemal A. (2015). Global cancer statistics, 2012. *CA Cancer J. Clin.* 65 87–108. 10.3322/caac.21262 25651787

[B21] Wang H. Huo X. Yang X. R. He J. Cheng L. Wang N. (2017). STAT3-mediated upregulation of lncRNA HOXD-AS1 as a ceRNA facilitates liver cancer metastasis by regulating SOX4. *Mol. Cancer* 16:136. 10.1186/s12943-017-0680-1 PMC5558651 28810927

[B22] Warburg O. (1956). On the origin of cancer cells. *Science* 123 309–314. 13298683 10.1126/science.123.3191.309

[B23] Weinberg F. Hamanaka R. Wheaton W. W. Weinberg S. Joseph J. Lopez M. (2010). Mitochondrial metabolism and ROS generation are essential for Kras-mediated tumorigenicity. *Proc. Natl. Acad. Sci. U S A.* 107 8788–8793. 10.1073/pnas.1003428107 20421486 PMC2889315

[B24] Wenner C. E. Spirtes M. A. Weinhouse S. (1952). Metabolism of neoplastic tissue. II. A survey of enzymes of the citric acid cycle in transplanted tumors. *Cancer Res.* 12 44–49. 14886961

[B25] Wilk A. Wyczechowska D. Zapata A. Dean M. Mullinax J. Marrero L. (2015). Molecular mechanisms of fenofibrate-induced metabolic catastrophe and glioblastoma cell death. *Mol. Cell Biol.* 35 182–198. 10.1128/mcb.00562-14 25332241 PMC4295376

[B26] Xu Y. Li Y. Jin J. Han G. Sun C. Pizzi M. P. (2019). LncRNA PVT1 up-regulation is a poor prognosticator and serves as a therapeutic target in esophageal adenocarcinoma. *Mol. Cancer* 18:141. 10.1186/s12943-019-1064-5 PMC6785865 31601234

[B27] Zeng H. Zheng R. Guo Y. Zhang S. Zou X. Wang N. (2015). Cancer survival in China, 2003-2005: a population-based study. *Int. J. Cancer* 136 1921–1930. 25242378 10.1002/ijc.29227

