# Supplementary figures and images for: LncRNA PPP1R14B-AS1 Promotes Tumor Cell Proliferation and Migration via the Enhancement of Mitochondrial Respiration
Source: Front Genet. 2020 Nov 11;11:557614. doi: 10.3389/fgene.2020.557614 (PMC7686783; doi:10.3389/fgene.2020.557614)

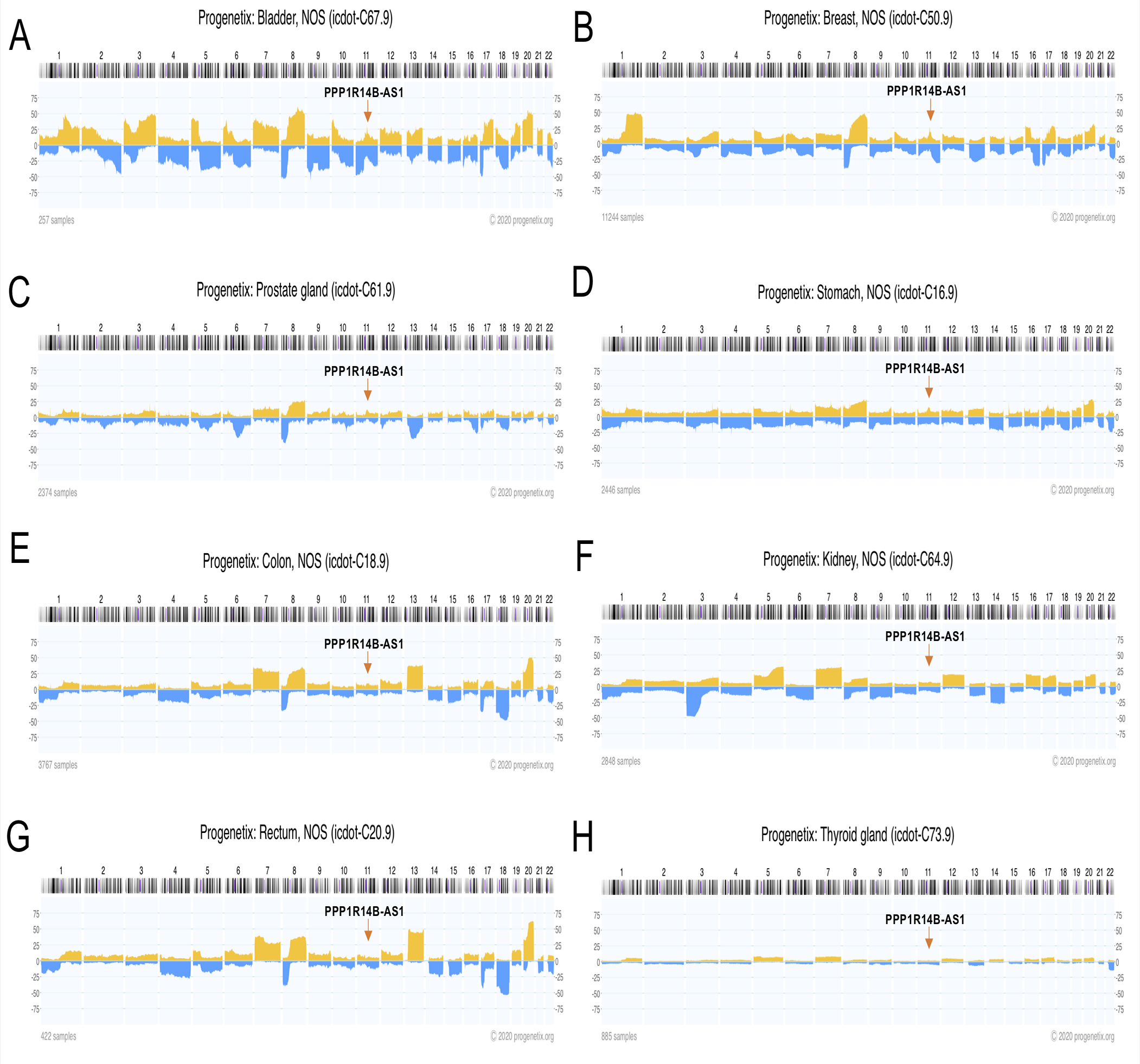

Supplement: Supplementary Figure 1 — The copy numbers of 11q13.1 across all chromosomes in different cancer samples. (A–E) Analysis of the copy numbers of PPP1R14B-AS1 across all chromosomes in 257 patients with BLCA, 11244 patients with BRCA, 2374 patients with PRAD, 2446 patients with STAD, and 3767 patients with COAD by Progenetix. (F–H) Analysis of the copy numbers of PPP1R14B-AS1 across all chromosomes in Kidney cancer patients, Rectum cancer patients and Thyroid gland cancer patients by Progenetix. [file Image_1.TIFF]

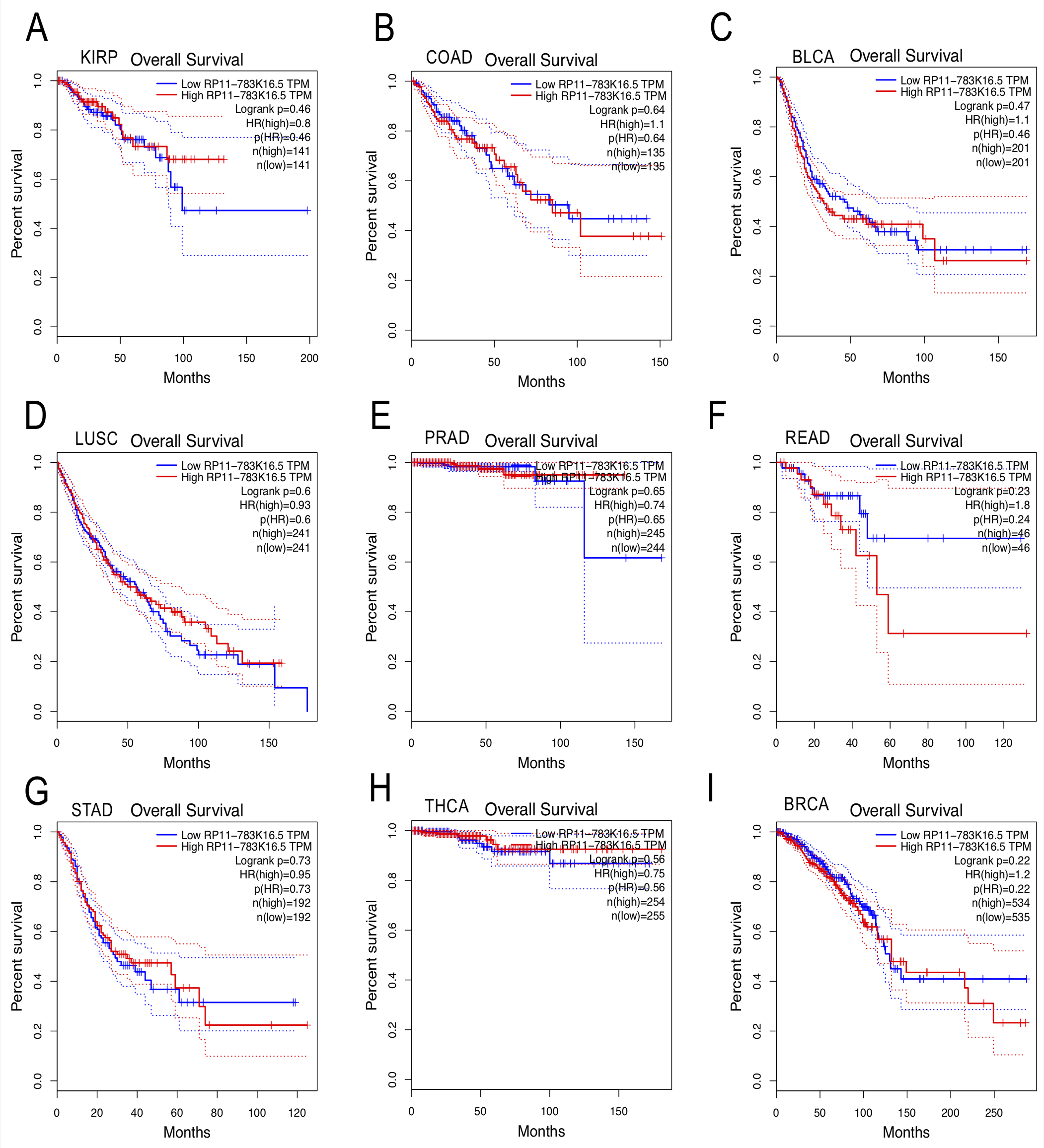

Supplement: Supplementary Figure 2 — PPP1R14B-AS1 upregulation showed no correlation with overall survival (OS) in several cancers. (A–I) Patients with KIRP (A), COAD (B), BLCA (C), LUSC (D), PRAD (E), READ (F), STAD (G), THCA (H), and BRCA (I) were divided into two groups in accordance with the median of the expression value of PPP1R14B-AS1 (PPP1R14B-AS1_low and PPP1R14B-AS1_high) respectively, and then Kaplan–Meier survival curves were constructed to analyze the OS of the two groups by GEPIA gene analysis tool (http://gepia.cancer-pku.cn/detail.php?gene=&clicktag=survival). Hazards Ratio (HR) was calculated based on Cox PH Model. [file Image_2.TIFF]
